# Effects of Intermittent Training on Anaerobic Performance and MCT Transporters in Athletes

**DOI:** 10.1371/journal.pone.0095092

**Published:** 2014-05-05

**Authors:** Grégoire Millet, David J. Bentley, Belle Roels, Lars R. Mc Naughton, Jacques Mercier, David Cameron-Smith

**Affiliations:** 1 ISSUL Institute of Sport Sciences University of Lausanne, Lausanne, Switzerland; 2 Department of Physiology, University of Lausanne, Lausanne, Switzerland; 3 Faculty of Health Science, University of Adelaide, Adelaide, South Australia, Australia; 4 ORION, Clinical Services Ltd, London, England; 5 Department of Sport and Physical Activity, Edge Hill University, Ormskirk, Lancashire, England; 6 Laboratoire de physiologie des Interactions EA 701, Institut de Biologie, Montpellier, France; 7 School of Nutrition and Exercise Sciences, Deakin University, Melbourne, Victoria, Australia; 8 Liggins Institute, University of Auckland, Auckland, New Zealand; Glasgow University, United Kingdom

## Abstract

This study examined the effects of intermittent hypoxic training (IHT) on skeletal muscle monocarboxylate lactate transporter (MCT) expression and anaerobic performance in trained athletes. Cyclists were assigned to two interventions, either normoxic (N; n = 8; 150 mmHg P_I_O_2_) or hypoxic (H; n = 10; ∼3000 m, 100 mmHg P_I_O_2_) over a three week training (5×1 h-1h30.week^−1^) period. Prior to and after training, an incremental exercise test to exhaustion (EXT) was performed in normoxia together with a 2 min time trial (TT). Biopsy samples from the *vastus lateralis* were analyzed for MCT1 and MCT4 using immuno-blotting techniques. The peak power output (PPO) increased (p<0.05) after training (7.2% and 6.6% for N and H, respectively), but VO_2_max showed no significant change. The average power output in the TT improved significantly (7.3% and 6.4% for N and H, respectively). No differences were found in MCT1 and MCT4 protein content, before and after the training in either the N or H group. These results indicate there are no additional benefits of IHT when compared to similar normoxic training. Hence, the addition of the hypoxic stimulus on anaerobic performance or MCT expression after a three-week training period is ineffective.

## Introduction

Traditionally the effects of intermittent hypoxic exposure or altitude training have been investigated on prolonged endurance performance. The proposed benefits of altitude training are an increase in red blood cell mass and haemoglobin concentration, which have the potential to enhance oxygen (O_2_) transport capacity and therefore endurance performance upon return to sea-level [17], [36], [42]. There is now considerable debate on the use of the different high-intensity hypoxic training methods as intermittent hypoxic training (IHT), which is frequent training of a higher intensity in hypoxia, or repeated sprints training in hypoxia (RSH) to improve endurance performance or repeated sprint ability in normoxia/at sea-level [8], [18], [25], [34], [35]. A variety of individual and team sports comprise a considerable anaerobic component [37]. The scientific rationale supporting the use of altitude training for anaerobic performance improvement is less compelling than that for the use of altitude training for the enhancement of endurance performance. However there is some evidence that altitude training can benefit sprinting or other short-term high-intensity performance that requires a substantial anaerobic metabolic contribution [2], [7], [9,]. However only few scientific studies have examined the effects of IHT on anaerobic performance [27].

One important physiological adaptation that may occur as a result of altitude training is an improvement in the capacity to buffer the exercise-induced increase in the concentration of hydrogen ions (H^+^) in skeletal muscle. Few studies have reported increase in buffering capacity following spring training in normoxia [17] as well with “living high – training high” [26] or “living high training low” [9] altitude methods. However, to date little is known about the effects of intermittent hypoxic stimulus on anaerobic performance.

The transport of lactate across the sarcolemma of skeletal muscle is mediated by proton-linked monocarboxylate transporters (MCT1 and MCT4) [12], [13], [16], [21], [30], [31], [43]. Due to the 1∶1 ratio between the lactate transport and the H^+^ transport, an increase in MCT1 and MCT4 could reduce the intracellular pH perturbations [13]. The MCT1 is expressed in the mitochondria [4] and facilitates the lactate influx into the muscle [13]. The MCT4 however, facilitates the removal of lactic acid and is mainly expressed in glycolytic fibers [13]. An increase in MCT1 and MCT4 protein expression is likely to decrease intracellular pH perturbations due to the coupled H^+^ transport [13]. Exercise training above VO_2_max in normoxic conditions is known to induce increases in both MCT1 and MCT4 content [2], [29].

However, there are limited studies investigating the effects of hypoxia on MCT expression in human skeletal muscle [5], [41]. In one study the authors concluded that 20 consecutive nights (9–10 h night^−1^) of hypoxic exposure (N_2_ enriched altitude house with simulated altitude at 2650 m, F_I_O_2_  = 16.3%), a ‘live high-train low’ (LHTL) scenario, decreased lactate production (as measured by stable isotope methodology) during intense exercise in well-trained athletes [5]. However, there was no effect of hypoxic exposure on MCT1 and MCT4 protein expression [5]. Aside from this investigation, there is little information concerning the effects of IHT on MCT expression in human skeletal muscle. The effects of IHT at high intensity on anaerobic performance in normoxia have also not been clearly established in trained athletes. The present study was therefore designed to evaluate the effects of acute intermittent hypoxic training on anaerobic performance and MCT expression, in human skeletal muscle. We tested the hypothesis that IHT would not provide larger MCT expression or anaerobic performance enhancement, when compared to the same similar training in normoxia.

## Methods

### Subjects

Eighteen male cyclists voluntarily participated in the study after giving written informed consent. The experimental procedures were approved by the institutional ethics committee (CPP Sud Méditerranée III, Faculty of Medicine, Nimes, France). All subjects were familiarized with the testing protocols and equipment used in the experiment. In addition, all the subjects were sea-level residents with no history of recent travel to altitude. After recruitment, the subjects were randomly allocated into two training groups, a Hypoxic group (H; n = 10) and a Normoxic group (N, n = 8). The subject characteristics for each group are presented in Table 1. The experiment took place in the pre-season prior to the main competition phase of the season.

**Table 1:** The subjects' physical characteristics of Normoxic (N: n = 8) and Hypoxic (H: n = 10) groups.

	*W0*		W4	
	N	H	N	H
Age (years)	24.2±0.4	24.4±0.3		
Height (cm)	181.6±0.03	180.1±0.5		
Weight (kg)	71.3±0.8	73.2±0.8	72.0±0.8	72.6±0.8

Mean ± SE; W0: pre-; W4: post-training.

### Experimental design

During the course of the experimental period (W1–W3), the subjects performed two interval-training and three continuous sessions per week in conjunction with their regular endurance training. Each training session included a 15 min warm-up and 15 min cool-down. The continuous training session consisted of 60 min at 60% of VO_2max_. The interval-training required the subjects to perform two sets of three repetitions each two min in duration and at an intensity of 100% Peak Power Output (PPO) [33]. Two min of rest was allowed between each repetition with 6 min rest between each set. Each session was performed at the same relative intensity specific to each condition (e.g. 100% PPO and 60% of VO_2max_ measured in normoxia or hypoxia for N or H, respectively) to ensure an equivalent training stimulus. The subjects trained on their own bicycle fixed on an electromagnetic resistant home-trainer (Elite Travel, Milan, Italy). Each session was meticulously controlled by the same researcher.

Training sessions were performed either in normoxic (P_I_O_2_ of 150 mmHg) or hypoxic (P_I_O_2_ of 100 mmHg, simulated altitude of ∼3000 m) environment for N and the H group, respectively. The average duration of the hypoxic stimulus per week during the training period was 382±8 min. No supplementary interval training was performed outside the supervised interval-training sessions.

Pre (W0) and post-training period (W4), a medical examination and determination of physical characteristics were completed (Table 1) and muscle biopsy samples were taken. The subjects performed an incremental exercise test to exhaustion in normoxic conditions. An identical incremental test to exhaustion under hypoxic conditions (P_I_O_2_ ∼100 mmHg) on a separate visit to the laboratory was also performed by the H group. An all-out exercise trial (∼2 min) was also performed under normoxic conditions by both groups to determine anaerobic performance. All the tests were randomized for both groups. A physician was in attendance at all times and was responsible for the safety of the subjects during the study. The weekly training that took place outside the experimental training was controlled.

### Environmental stimulus

The hypoxic environment (equivalent to ∼3000 m) was induced by the continued delivery of a hypoxic gas mixture by a system that modifying nitrogen content and therefore decreased the inspired O_2_ fraction (Altitrainer 200, SMTEC, Geneva, Switzerland). This device allows the production of large quantities of hypoxic gas mixture, up to 200 L^.^min^−1^, with an easily adjustable O_2_ fraction over a large range and with a short response time. Air inhaled from outside the machine is controlled with a fixed quantity of nitrogen from a bottle which is then mechanically mixed prior to being stocked in a buffer tank of 30 L. The first safety check is ensured by the mixer's mechanical limit, which cannot exceed a certain nitrogen fraction (F_I_O_2_  = 9.7%). The user inhales the mixture contained in the tank through a Hans Rudolph two-way respiratory valve. An O_2_ probe, a second safety check, plunged in the buffer tank, measures the PO_2_. This probe, with aid of a microprocessor, allows the PO_2_ of the inhaled mixture, or the equivalent altitude to be displayed and cannot decrease less than 66 mmHg (5500 m). If necessary, the user can constantly modify the composition of the air which they breathe and thus change the simulated altitude. A breath-by-breath analyser was attached to the system in order to measure respiratory exchange.

### Performance Tests

The subjects completed two different performance tests before (W0) and after (W4) the training.

#### 1) Incremental test to exhaustion

The subjects performed an incremental test to exhaustion to determine the maximal oxygen uptake (VO_2max_; ml^.^kg^−1.^min^−1^), the peak power output (PPO; W), maximal heart rate (HR_max_; bpm), and maximal ventilation (VE; l^.^min^−1^). The test began with a three min stage at an initial power output of 60 W and than the workload progressively increased by 30 W every minute until exhaustion. Exhaustion was reached when two out of three of the following criteria were obtained: 1) heart rate (HR) approaching an age predicted maximum value (220 - age); 2) a plateau in VO_2_ despite an increase in exercise intensity; and 3) a RER >1.1. PPO was defined as the highest mechanical power maintained during one min.

In addition, only the H group performed the same test also under hypoxic conditions on a separated laboratory visit and prior the start of the training protocol and biopsy (data not presented in this paper). This test was necessary to provide data to determine the workloads (100% PPO and 60% of VO_2max_) under hypoxic conditions for the training sessions for the H group.

#### 2) All-out exercise test

The all-out exercise test consisted of cycling at the highest power output to exhaustion, approximately 2 min, in normoxic conditions. This test is a reliable measurement for anaerobic performance [22]. This test assessed the subject's anaerobic capacity expressed as maximal accumulated oxygen deficit (MAOD) [23], the peak oxygen consumption (VO_2peak_, ml^.^kg^−1.^min^−1^), average power output (P_aver_, W) and maximal lactate (La_max_; mmol^.^L^−1^) accumulation were measured.

### Physiological parameters

Respiratory gas exchange was measured pre and post intervention using a K4^b2^ (Cosmed, Rome, Italy). Prior to each test, the system was calibrated according to manufacturer's recommendations. Breath-by-breath data were reduced to 30 s averages and VO_2max/peak_ was determined as the highest 30 s VO_2_ average. Heart rate (HR) was constantly recorded by the means of a HR monitor (S810, Polar, Kempele, Finland) integrated to the Cosmed system.

The accumulated oxygen (AO_2_) deficit was calculated as the difference between the AO_2_ demand and the AO_2_ uptake measured during the all-out exercise test [23]. The AO_2_ demand was taken as the product of the O_2_ demand and the duration of the exercise, assuming that the O_2_ demand was constant throughout the whole exercise period. The AO_2_ deficit calculated was reported as the “maximal AO_2_ deficit” (MAOD) for cycling [22], [23]. Changes in MAOD determined after the training period were reported as the percentage increase, calculated from the pre-test values (W0).

All the tests were performed on a bicycle equipped with an SRM road professional powermeter (Schoberer Rad Messtechnik, Jülich, Welldorf, Germany). The saddle height on the cycle ergometer was kept identical for all the tests. Power output and pedalling cadence were recorded every second and 30-s averaged values were stored. The calibration procedure and technical aspects concerning SRM crank system have previously been described in detail. The SRM road professional powermeter is constructed with four strain gauges and shown to have a high accuracy in power measurement. The 95% limits of agreement is 2.1 W which is equivalent to 1.8% [11]. After each test the rate of perceived exertion (RPE) was recorded.

### Training outside experimental design

The training completed outside the experimental design was recorded daily using a computerized training diary during the three weeks of training period. The type of activity, *i.e*. cycling, swimming, running, and the intensity were recorded, with training intensity divided into five intensity levels [28]. All training sessions outside the protocol were individually timed and each exercise categorized according to the five intensity levels. The performed training duration was multiplied by its corresponding multiplying factor, *i.e*. 2, 4, 6, 10, and 16, respectively and the sum was then divided by the overall duration of the session to calculate the average intensity of each training session. It was deemed not valid to compare hourly volume as the subjects performed exercise training in different modes (swimming, running).

### Muscle biopsy

One week after all cycling sessions, muscle samples were obtained from the *vastus lateralis* using the percutaneous needle biopsy technique after administration of local anesthesia (xylocaine) as previously described and performed in our laboratory [38], [39]. The biopsies were taken by the same researcher from the same site, *i.e*. in the middle of the line between *spina iliaca* anterior superior and the upper outer corner of the *patella* at a depth of 1.5 - 2.0 cm from the fascia in all the subjects. The muscle samples were immediately frozen in liquid nitrogen and stored at −80°C until further analysis.

### Western Blotting

Whole muscle protein was isolated from each sample by a method previously described and previously used in our laboratory [6], [39]. Muscle protein concentration was determined in duplicate by bicinchoninic acid assay (Pierce, Interchim, Montluçon, France) with the use of BSA as a standard.

Affinity polyclonal antibodies directed against the carboxy terminus of human MCT1 and MCT4 were produced with the synthetic peptide C-Ahx-KDTEGGPKEEESPV-OH for MCT1 and C-AHX-GEVVHTPETSV-OH for MCT4, like the sequence used in other research work [4], [5], [7], [18]. Western Blotting was probed with these antibodies and Chemicon International antibodies (Temecula, California, USA. Rabbit anti-MCT1: AB3538P and rabbit anti-MCT4: AB3316P). Polyclonal antibodies yielded a single band on a Western blot that corresponded to 43 kD, consistent with the molecular mass reported earlier [21]. Antibody specificities were confirmed in preliminary experiments in which the peptides blocked the detection of MCT1 and MCT4. Samples of muscle homogenates (12 µg protein) and pre-stained molecular mass markers (Bio-Rad), were separated on 10% Bis-Tris-acrylamide gels (200 V for ∼60 min) with the Novex system (Invitrogen, Groningen, The Netherlands). Proteins were then transferred from the gels to polyvinylidene difluoride (PVDF) membranes (30 V, 180 min), and the membranes were incubated on a shaker overnight at 4°C temperature in buffer D (20 mmol.l^−1^ Tris base, 137 mmol.l^−1^ NaCl, 0.1 mol.l^−1^ HCl, adjusted to pH 7.5, 0.1% [vol/vol] Tween 20, and 5% [wt/vol] non-fat dried milk). The membranes were then incubated with diluted carboxy-terminal of either MCT1 antibody (1∶90 000) or MCT4 (1∶90 000) in buffer D for 2 hours at room temperature, followed by 4 washes (4×5 min washes) in buffer E, *i.e*., buffer D without dried milk and then incubated for 50 min with goat anti-rabbit immunoglobulin G horse-radish peroxidase-conjugated secondary antibody (1∶10 000, BI2407, BioSys, Compiègne, France) in buffer E. Membranes were washed as previously described and MCT1 or MCT4 expression was detected by ECL (Biomax MR films, Kodak, Reuil-Malmaison, France). The identified bands were developed and fixed using a Hyperprocessor (RNP 1700, Amersham, Les Ulis, France). MCT1 and MCT4 protein band densities determined by scanning the blots on a scanner (AGFA Duo Scan T1200, New York, USA) and Scion Image software (Scion Corp, Frederick, MD). Results were expressed in arbitrary optical density (OD) units (a.u.) as previously [6], [38], [39].

### mRNA analysis

Total cellular RNA was extracted as previously described [40]. The RT-PCR was performed using the Applied Biosystems 7500 Real Time PCR Machine (Applied Biosystems, Foster City, CA). PCR was performed in duplicate with reaction volumes of 20 µl, containing SYBR Green 1 (Applied Biosystems, Foster City, CA), forward and reverse primers and cDNA template (diluted 1∶20). Data were analyzed using a comparative critical threshold (Ct) method where the amount of target normalized to the amount of endogenous control relative to control value is given by 2^−ΔΔCt^. The efficacy of *cyclophilin* as an endogenous control was examined using the equation 2^−ΔCt^. It was considered an appropriate control for this study when no changes in the expression of the gene were observed (data not shown) [19]. Primers were designed using Primer Express software package version 3.0 (Applied Biosystems, Foster City, CA) from gene sequences obtained from GenBank. Primers were designed spanning intron-exon boundaries to prevent amplification of the target region for any contaminating DNA. Primer sequence specificity was also confirmed using BLAST. A melting point dissociation curve was generated by the PCR instrument for all PCR products to confirm the presence of a single amplified product.

### Statistical analysis

All values are reported as mean ± standard error (SE). A power calculation was conducted for the primary outcome measure of average power in the time trial for the initial 20 subjects (10 per group) recruited. The calculation (using PASS 11) was based upon unpublished test-retest reliability data from our laboratory to produce an effect size of 0.72 and a power of 86% [10]. Since we had two subjects withdraw, a re-calculation of power indicated that, eight subjects in the control group and 10 in the experimental group gave 81% power to detect a standardised effect size of 0.72 for the condition-by-time interaction.

After analysis of the normality and the homogeneity of variance of the tested samples, the influence of the two training methods on the measured variables was analyzed using a two-way (training group × time) analysis of variance (ANOVA) with repeated measures on the second factor. A student Newman-Keuls post-hoc test was used to analyze significant effects. All analyses were undertaken using SigmaStat 2.3 (Jandel Corporation, San Rafael, CA). Statistical significance was accepted at P<0.05.

## Results

**Figure 1:**
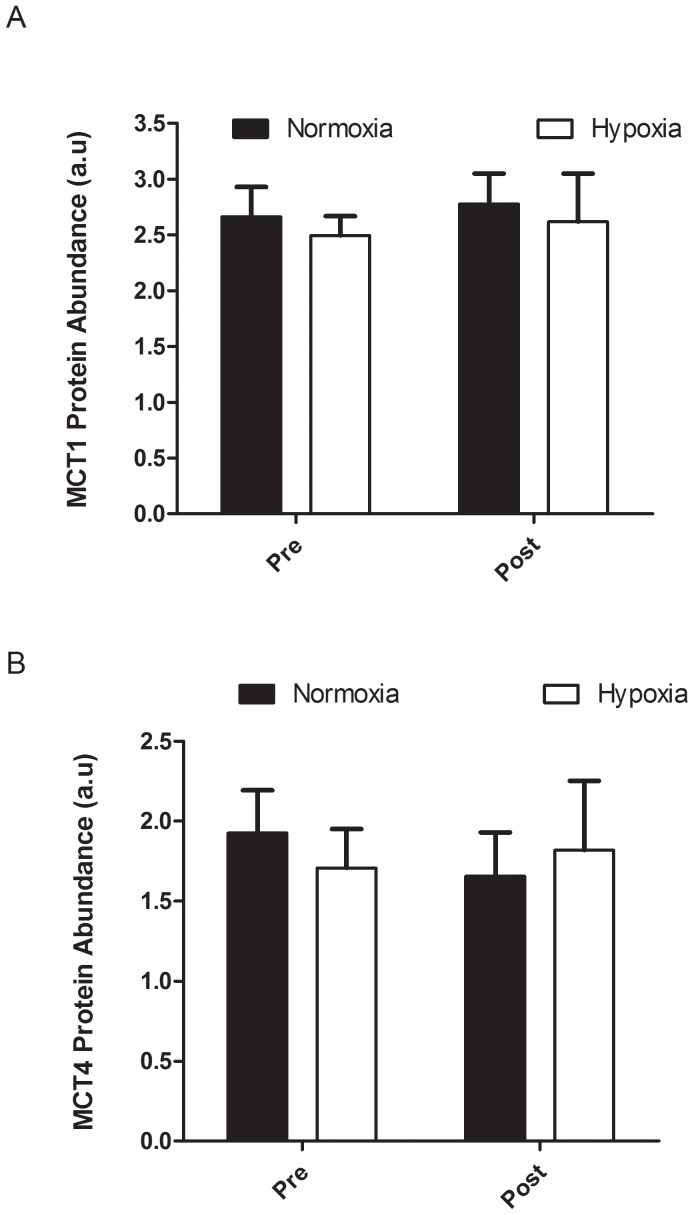
The MCT1 (A) andMCT4 (B) protein content for the normoxic (N; n = 8) and hypoxic (H; n = 10) groups, pre (W0) and post (W4) the 3 wk IHT period.

**Figure 2:**
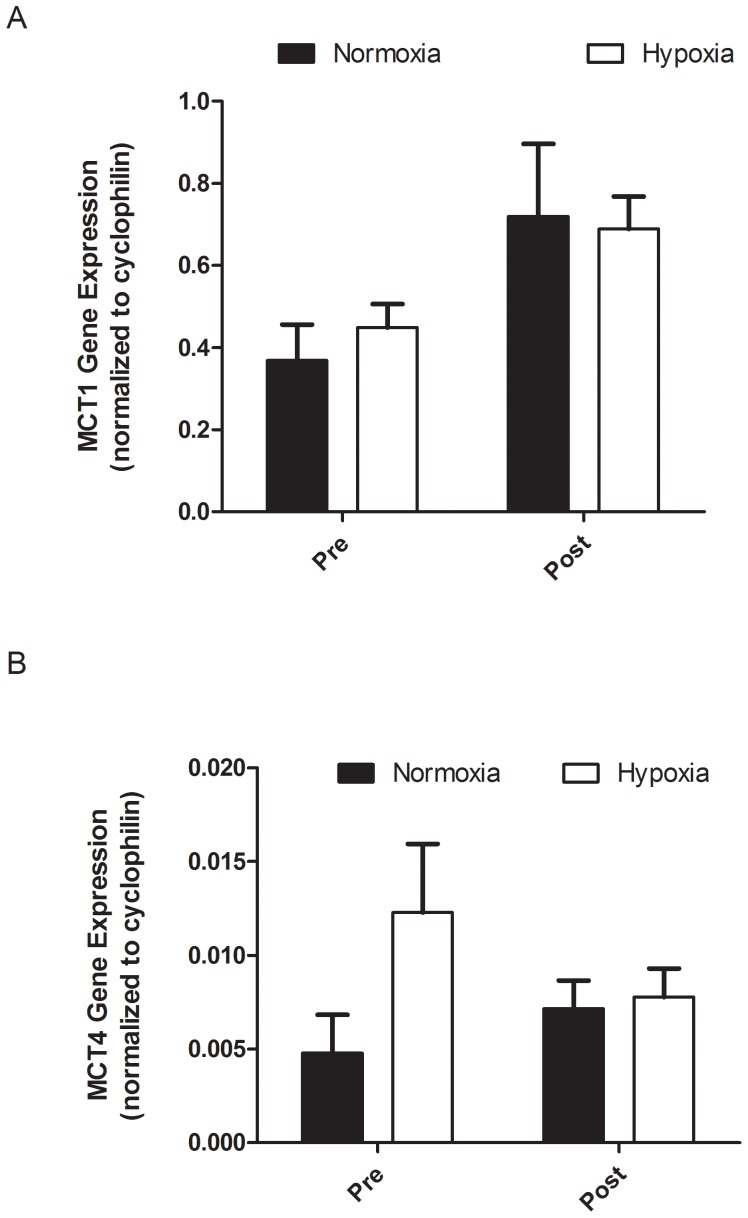
The MCT1 (A) and, MCT4 (B) mRNA for the normoxic (N; n = 6) and hypoxic (H; n = 8) groups, pre (W0) and post (W4) the 3 wk IHT period.

Table 2 presents the workloads of each group averaged over the three weeks for the interval training and continuous workload sessions. No significant differences in (relative) training workloads in- and outside the training sessions were observed between the groups (Table 3). No significant differences were observed in PPO or VO_2_max obtained during the incremental exercise test or performance during the 2 min all-out trial values before the training period between the two training groups (Table 4). PPO significantly (p<0.01) increased with training but this increase was not different between training groups (7.2% and 6.6% for N and H, respectively). No significant improvement was found for VO_2_max in the two training groups. Both groups significantly (p<0.01) improved performance (P_aver_) during the 2 min all-out exercise trial (7.3 and 6.4% for N and H group, respectively). The H group showed a significant decrease (P = 0.006) of −5.1% in VO_2_peak measured during the 2 min all-out test between W0 and W4 (Table 5). The N group showed no significant difference in VO_2peak_ (−2.4%) between W0 and W4 (Table 5). No statistical differences were found in muscle MCT1 and MCT4 protein and mRNA content after each training intervention (Figure 1 and 2). Furthermore, we did not find any relationship between MCT content and anaerobic performance.

**Table 2:** Averaged percentage (Mean ± SE) of target power output (target PO), *i.e*. 100% PPO for interval sessions and 60% of VO_2max_ and the averaged absolute power output (W_absolute_; W) for continuous training sessions, of Normoxic (N, n  =  8) and Hypoxic (H; n  =  10) groups.

	Continuous		Interval	
	% of target PO	W_absolute_	% of target PO	W_absolute_
N	91.7±0.6	185.0±7.5	91.0±0.7	310.9±4.5
H	91.8±1.6	130.0±0.3	91.8±1.0	259.4±0.3

**Table 3:** Mean (± SE) intensity of additional training outside the experimental design of each training week for Normoxic (N; n  =  8) and Hypoxic (H; n  =  10) groups.

	W1	W2	W3
N	3.1±0.05	3.1±0.06	3.2±0.1
H	2.9±0.09	3.0±0.05	3.0±0.07

W1: first week; W2: second week; W3: third week of training protocol.

**Table 4:** Mean (± SE) values of the incremental test to exhaustion of Normoxic (N; n = 8) and Hypoxic (H; n = 10) groups.

	N		H	
	W0	W4	W0	W4
VO_2max_(ml^.^kg^1^.min^−1^)	58.1±0.8	61.0±1.2	58.5±0.7	58.3±0.6
PPO (W)	341.7±3.5	366.3±3.2*	339.0±0.5	361.5±4.4*
HR_max_ (bpm)	190.1±1.1	188.3±1.5	189.7±1.1	189.4±1.0
VE_max_ (L^.^min^−1^)	159.9±2.6	174.3±2.5	154.2±1.4	155.0±1.7
RPE_max_	17.9±0.2	17.9±0.1	16.4±0.2^§^	16.9±0.2

W0: pre-; W4: post-training; VO_2max_: highest value of the oxygen consumption averaged over 30 s; PPO: peak power output; VE_max_: highest value of ventilation averaged over 30 s; RPE: rate of perceived exertion.

*P<0.05 for the differences within a group versus W0; ^§^P<0.05 for the differences between groups at a matched time point.

**Table 5:** Mean (± SE) values of the 2-min all-out exercise of Normoxic (N; n = 8) and Hypoxic (H; n = 10) groups.

	N (n = 8)		H (n = 10)	
	W0	W4	W0	W4
P_aver_ (W)	377.6±3.4	405.3±2.5*	345.5±5.3	367.6±5.3*
VO_2peak_ (ml^.^min^−1.^kg^−1^)	57.5±0.6	56.1±0.7	56.7±0.6	53.8±0.6*
HR_max_ (bpm)	178.7±1.4	178.0±1.2	186.6±1.0	181.8±0.9
VE_max_ (L^.^min^−1^)	156.8±2.0	177.2±3.0	150.9±1.8	144.3±1.4^§^
RPE_max_	17.7±0.3	18.4±0.2	16.8±0.2	16.3±0.2^§^
La_max_ (mmol^.^L^−1^)	15.1±0.2	16.6±0.3	14.8±0.3	14.6±0.3
MAOD (ml^.^kg^−1^)	32.7±1.0	35.4±1.1	31.8±1.0	34.3±0.1

W0: pre-; W4: post-training; P_aver_: average power output; VO_2peak_: highest 30 s VO_2_ average; HR: highest value of heart rate averaged over 30 s; VE_max_: highest value of ventilation averaged over 30 s; RPE: rate of perceived exertion; La_max_: maximal lactate; MAOD: maximal accumulated oxygen deficit.

*P<0.05 for the differences within a group versus W0; ^§^P<0.05 for the differences between groups at a matched time point.

## Discussion

This study was conducted to examine the effects of IHT (compared to conventional interval-training in normoxia) on anaerobic performance and MCT expression in human skeletal muscle. The key findings of the study were that PPO at sea-level increased with training regardless of whether this was performed in hypoxia or normoxia. Furthermore, anaerobic exercise performance at sea-level increased to a similar extent after the hypoxic and normoxic training period. In addition, neither the hypoxic nor normoxic training had any effect on MCT1 and MCT4 protein content. Hence, these results indicate there are no additional effect of the hypoxic stimulus on anaerobic performance after a three-week training period.

The PPO and all-out exercise performance improved after three weeks of training. This suggests that three weeks of training consisting of two interval training sessions and three steady work training sessions are sufficient to obtain significant improvements in sea-level aerobic and anaerobic performance. Meanwhile, neither VO_2max_ nor VO_2peak_ enhanced after the training period in well-trained athletes. The present results are in accordance with a previous study where the authors evaluated a lower intensity hypoxic training of 105 min of cycling training at 60 to 70% of HR reserve a day during 10 consecutive days at 2500 m in eight triathletes [24]. After the training protocol, VO_2max_ was unchanged in both groups indicating that performance can be improved without complimentary changes in VO_2_max. However, PPO significantly increased with 6% and also significant improvements in anaerobic power (5%) and anaerobic capacity (4%) measured during a 30-s Wingate test were observed in the IHT group. The authors concluded that 10 days of ∼2 hours a day of IHT did not induce changes in VO_2max_ of elite triathletes, but did enhance anaerobic power and capacity [24]. An enhanced H^+^-buffering capacity has been shown to be associated with the improvement in anaerobic/sprint performance following spring training in normobaric conditions. It has been reported in Danish national team cross-country skiers who lived at 2100 m and trained at 2700 m for 14 days, a significant 29% increase in maximal oxygen deficit, as well as a significant 6% increase in the buffering capacity of the *gastrocnemius* muscle [26]. However, as there was no sea-level control group data in this study, it is therefore difficult to draw definitive conclusions regarding the findings. Others [9]found a significant 18% increase in *in-vitro* muscle buffer capacity (βm) of the *vastus lateralis* in male athletes after 23 days of living high-training low at a simulated altitude of 3000 m. These authors [9] suggested therefore that the chronic hypoxic stimulus per se increased the βm.

The observed increase in the aerobic and anaerobic performance without any changes in VO_2max_ and VO_2peak_ could also be explained by a decrease in the rates of lactate appearance (R_a_) and disappearance (R_d_). Whilst lactate kinetics during exercise were not measured during this study, others [5] have investigated the effect of 20 nights of 9–10 h of LHTL at a simulated altitude of 2650 m, and found an decrease in whole body R_a_ during 30 min exercise, at 85% VO_2peak_, in well-trained athletes. Meanwhile, these authors [5] investigated the effect of hypoxia during rest and/or sleeping in contrast with the present study where training rather than rest and/or sleeping was performed under hypoxic conditions. Moreover, the buffering capacity and pH regulation did not show any changes after the 20 nights of LHTL [5]. Therefore, the observed increase in PPO and anaerobic exercise performance are likely due to mechanisms independent of lactate metabolism, *i.e*. buffer capacity and MCTs.

The present work is one of the first studies to examine change in anaerobic performance with IHT. The results show that whilst IHT can be beneficial for performance of 2 min of all-out exercise, the effect does not have any additional benefits than conventional training in normoxia. Whilst these results suggest that IHT should only be reserved for athletes aiming to improve performance in events with a considerable aerobic component, it should be considered that performance at altitude could be improved. A previous study showed that work performance in hypoxia was improved after a period of training in hypoxic conditions [33]. Moreover, there were no differences between or within both groups for citrate synthase and hydroxyacyl-CoA-dehydrogenase activity [33]. Since anaerobic performance is strongly associated to high lactate transport/oxidation, it is of interest that we did not observe any significant changes in the MCT protein expression. In other words, three weeks of IHT does not result in an up-regulation of MCT protein, and is in accordance with previous findings [5]. These authors did not observe an effect of hypoxic exposure, *i.e*. 20 nights of LHTL on MCT1 and MCT4 protein density. Similarly, other authors found no changes in MCT1 and MCT4 content in the *vastus lateralis* muscles after eight weeks at natural altitude of 4100 m [14]. In contrast, other researchers [20] observed that in female Wistar rats, after eight weeks in a hypobaric hypoxic environment (4300 m), a similar decrease of 47% in MCT1 and MCT4 of in the *plantaris* muscle. Moreover, these authors found also a tissue-specific effect of hypoxic training on the protein expression [20]. Therefore, as suggested, human muscle could also experience similar fibre-type-dependent changes which in the *vastus lateralis* muscle - as analyzed in the present study - due to its mixed fibre-type composition, could have been overwritten [15].

The lack of any change in MCT1 and MCT4 expression could be due to the training intensity in the present study not of a sufficient magnitude to induce changes in MCT protein content. Indeed, some authors have suggested that the changes in MCT protein expression are related to the training intensity [3]. These authors suggested that MCT1 and MCT4 protein content might increase after an intense anaerobic exercise training, as has been previously observed [30] and that only MCT1 protein content might increase after an aerobic training as they observed [3]. After three weeks of chronic muscle stimulation of 24 h/day in red *tibialis anterior* muscle, MCT1 protein content was significantly increased by 78%, whereas MCT4 protein expression was not altered [3]. However, after eight weeks of one-legged knee-extensor high-intensity exercise training in healthy male subjects, the MCT1 and MCT4 protein content had increased significantly by 76 and 32% respectively [30]. In addition, it has been shown that after seven to eight weeks of one-legged knee-extensor exercise training at an intensity of 150% of thigh VO_2max_, the MCT1 protein content increased with 115% (P <0.05), whereas no significant difference was observed for the MCT4 protein content (+111%, P>0.05) [14]. Whilst this study demonstrates supramaximal exercise training may evoke large increases in MCT1 and MCT4 expression, some precaution should be taken when interpreting these results, as the study design involved single-leg knee-extensor exercise training which might not induce the same physiological adaptations compared to the present study where “whole body” cycling exercise training was incorporated. Even if a strong point of the present study was that the relative exercise intensity (e.g. 91–92% of PPO – Table 2) during interval-training was similar in normoxia and in hypoxia, this intensity and the duration of the work-interval (e.g. 2-min) were likely not optimal for inducing a high lactate production. It was reported that the increase in lactate/H+ transport capacity is more likely in response to the high-intensity training than the hypoxic condition per se [16]. Therefore, one may assume that the present training intensity was not enough ‘glycolytic’ for inducing an increase in MCTs in any group. In addition, MCT4 was reported more sensitive than MCT1 to hypoxia since after chronic hypobaric hypoxia exposure [32] or extreme hypoxic conditions [41], only MCT4 protein or mRNA levels increased. In the present study, the low hypoxic dose (combination of a total exposure <20 h and a moderate normobaric hypoxia level; e.g. simulated altitude of 3000 m) explains probably why neither MCT4 nor MCT1 were increased.

In conclusion, three weeks of intermittent hypoxic training increased incremental exercise performance at sea-level. At the same time, anaerobic performance was enhanced without altering the expression of MCT1 and MCT4 protein content. However, no additional benefits of training in hypoxia were observed when compared to similar high-intensity training in normoxia. Therefore, IHT at intensity at or close to PPO as a technique to further improve anaerobic performance at sea-level is questionable.

## Impact and Perspective

Scientists, coaches and athletes should have a better understanding of the role that hypoxia plays in training for anaerobic exercise or indeed training for such intermittent high intensity performance. No additional adaptations or anaerobic performance enhancement should be expected above that which athletes can accomplish at sea-level.
